# Determinants of patients' satisfaction with health-care services: the role of patient demographics and individual factors

**DOI:** 10.4314/ahs.v25i4.23

**Published:** 2025-12

**Authors:** Faith Wangombe, Violet Maritim, Mary Amatu

**Affiliations:** 1 Mount Kenya University, School of Public Health; 2 Mount Kenya University-Thika Campus, School of Public Health; 3 Meru University of Science and Technology, School of Health Sciences

**Keywords:** Patients' satisfaction, health-care services, patient demographics, individual factors

## Abstract

**Background:**

Patient satisfaction is a fundamental measure of healthcare quality and service responsiveness. In Kenya's public health system, understanding patient experiences is essential for enhancing care delivery under the UHC agenda. This study examined the influence of socio-demographic and individual factors on patient satisfaction.

**Methods:**

A cross-sectional descriptive design was employed involving 277 adult patients selected through simple random sampling. Data were collected via a questionnaire and analyzed using SPSS version 27. Descriptive statistics summarized patient characteristics, while chi-square tests and binary logistic regression identified associations and predictors of satisfaction (p < 0.05).

**Results:**

Overall, 49.8% of respondents reported being satisfied, while 24.6% were dissatisfied. Employment status showed a borderline significant association with satisfaction (*χ*^2^ = 21.00, p = 0.050), whereas age (p = 0.855), gender (p = 0.687), income (p = 0.088), education (p = 0.740), and marital status (p = 0.875) were not significant predictors. Among individual-level factors, effective communication with providers was the strongest predictor of satisfaction (B = 0.857, p = 0.0008, OR = 2.357). Other variables such as trust (B = 0.236, p = 0.140), waiting time (B = 0.191, p = 0.079), affordability (B = -0.121, p = 0.419), and understanding of treatment (B = -0.163, p = 0.196) were not statistically significant.

**Conclusion:**

Patient satisfaction was moderate and influenced more by interpersonal interactions than demographic profiles. Communication quality emerged as the most important determinant. Enhancing relational aspects of care through communication training and routine feedback systems may improve satisfaction and align public health services with patient-centered care goals.

## Introduction

Patient satisfaction has been widely acknowledged as a key measure of healthcare quality and a critical outcome in evaluating health systems globally^1^,^2^. It reflects the extent to which healthcare services meet or exceed patient expectations and provides valuable feedback for improving clinical care, facility management, and health policy^3^. In both developed and developing contexts, satisfied patients have been more likely to adhere to treatment regimens, continue using healthcare services, and recommend them to others^4^. Consequently, measuring and understanding patient satisfaction has become a core component in the assessment of service delivery effectiveness, particularly in primary and secondary care institutions^5^.

Various factors have been associated with patient satisfaction, including individual socio-demographic characteristics, perceptions of care, and structural attributes of health facilities^6^,^7^. Age, gender, income level, education, and employment status have all been explored as potential determinants, though findings across contexts have often varied in significance and strength^8^,^9^. In many resource-limited settings, particularly in Africa, patient satisfaction has also been influenced by health system challenges such as limited staff capacity, resource shortages, and service delays^6^,^10^. These challenges highlight the importance of contextualized research that considers both systemic and user-level factors within a specific healthcare environment.

Health system reforms in Kenya have emphasized equitable access, quality improvement, and community-centered care as part of national goals toward achieving Universal Health Coverage^11^–^13^. Despite infrastructure and service delivery expansions, concerns about patient experience and satisfaction in public hospitals have persisted, especially in high-demand urban and peri-urban areas^6^,^14^. Devolved health governance has provided counties with the autonomy to tailor services to their population's needs; however, this decentralization has also revealed variations in service quality and user perceptions across regions^3^,^15^,^16^. The need to better understand how patients perceive public healthcare services in specific counties has therefore become increasingly urgent for local health system improvement.

This study aimed to assess the determinants of patient satisfaction with healthcare services in Level 5 public hospitals in Kiambu County, Kenya. It examined the influence of socio-demographic and individual patient characteristics on patient satisfaction. By focusing on county referral hospitals within the Kenyan health system, the study sought to contribute empirical evidence for quality improvement initiatives and inform patient-centered health policy at both the local and national levels.

## Methods

### Study Design and Setting

This study employed a cross-sectional descriptive design to assess patient satisfaction and its determinants in public healthcare services. The design was appropriate for collecting data at a single point in time to capture patients' experiences and perceptions without requiring longitudinal follow-up, making it suitable for evaluating service quality in a busy clinical environment^17^. The research was conducted in three Level 5 public hospitals located in Kiambu County, Kenya. The county, situated in the central region of Kenya, spans approximately 2,449 square kilometers and hosts a population of over 2.4 million as of the 2019 census. Characterized by both urban (60%) and rural (40%) populations, Kiambu County has a dynamic socio-economic profile, which contributes to high demand for public health services^18^. The study sites were selected due to their high patient volumes, capacity to offer specialized care, and their strategic role as referral centers within the public healthcare system.

### Study Population and Sampling Procedure

The study population comprised adult patients (aged 18 years and above) attending outpatient clinics in the selected Level 5 hospitals during the study period. These facilities serve both local residents and referrals from lower-tier health institutions. A simple random sampling method was employed to ensure that all eligible patients had an equal chance of selection, thereby minimizing selection bias and increasing representativeness^19^. Patients were eligible if they were physically and mentally capable of participating, provided informed consent, and were present in the outpatient department on the day of data collection. This inclusive approach enabled a diverse and reflective sample of public hospital users in Kiambu County.

### Data Collection Instrument and Procedures

Data were collected using a structured, interviewer-administered questionnaire designed to assess socio-demographic characteristics, individual experiences, institutional service factors, and satisfaction with outpatient care. A five-point Likert scale was used to capture patient satisfaction, ranging from “Extremely Satisfied” to “Extremely Dissatisfied.” The questionnaire underwent a pilot study involving 10% of the intended sample, which facilitated refinement for clarity, language, and structure. Content validity was established through expert review and participant feedback, while internal consistency was confirmed with a Cronbach's alpha of 0.78 20. Data collection was conducted face-to-face in outpatient waiting areas. The principal investigator explained the study purpose, ensured voluntary participation, and obtained written informed consent. Participants either completed the questionnaire independently or with assistance, depending on literacy and preference. The researcher remained available to clarify queries without influencing responses. Completed forms were reviewed for completeness and securely stored before data entry.

### Data Management and Analysis

Data entry and analysis were performed using the Statistical Package for the Social Sciences (SPSS) version 27. Descriptive statistics—including frequencies and percentages—were used to summarize participants' characteristics and satisfaction levels. Inferential statistics were applied to examine associations between patient satisfaction and selected variables. Chi-square tests were used to assess relationships between categorical variables, while binary logistic regression was employed to identify significant predictors of satisfaction. The level of significance was set at p<0.05 for all inferential analyses. Findings were presented using tables and narrative summaries in alignment with the study objectives.

## Ethical Considerations

Ethical approval was obtained from the Mount Kenya University School of Graduate Studies and Ethics and Research Committee, the County Health Ethics and Research Committee, the National Commission for Science, Technology, and Innovation (NACOSTI), and the administrations of the three participating Level 5 hospitals. All participants provided written informed consent prior to data collection. Ethical principles such as voluntary participation, confidentiality, anonymity, and the right to withdraw at any stage were strictly upheld throughout the research process

## Results

### Level of Satisfaction

The results of overall patient satisfaction with healthcare services among those seeking care at Level 5 public hospitals in Kiambu County show 49.8% of the respondents were either ‘satisfied’ or ‘very satisfied,’ with 45.1% indicating they were satisfied and 4.7% very satisfied. Meanwhile, 24.6% of respondents were either dissatisfied or very dissatisfied, while 15.2% were unsure of their satisfaction (Table 1).

**Table 1:** Overall Satisfaction Levels Reported by Participants

Satisfaction level	Frequency	Percent (%)
Very dissatisfied	13	4.7%
Dissatisfied	55	19.9%
Not sure	42	15.2%
Satisfied	125	45.1%
Very satisfied	13	4.7%
**Total**	**277**	**100%**

### Demographic Characteristics and Their Association with Patient Satisfaction

Most respondents were aged 25–39 years (39.7%), followed by 45–55 years (25.3%), under 25 (21.7%), and over 55 (13.4%). Nearly half were self-employed (46.9%), while 33.2% were unemployed. The majority earned below Ksh 20,000, with only 6.5% earning above Ksh 50,000. Over half were married (56%), and 88.5% lacked social support. Chi-square analysis showed a borderline significant association between employment status and satisfaction (p = 0.050), indicating financial stability may enhance satisfaction. However, age, gender, education, income, marital status, and social support showed no significant relationships (p > 0.05). These findings suggest that while socio-demographic factors may shape healthcare experiences, they do not independently predict satisfaction in this public outpatient setting (Table 2).

**Table 2:** Demographic Characteristics and Their Association with Patient Satisfaction

Variable	Category	Frequency (n)	Percentage	Chi-square (X^2^)	p-value
**Age**	Below 25 years	60	21.7%	7.04	0.855
	25–39 years	110	39.7%		
	45–55 years	70	25.3%		
	Above 55 years	37	13.4%		
**Gender**	Male	130	46.9%	2.94	0.687
	Female	147	53.1%		
**Employment Status**	Casual	30	10.8%	21.00	0.050
	Self-employed	130	46.9%		
	Permanently employed	25	9.0%		
	Unemployed	92	33.2%		
**Income Level**	Less than	107	38.6%	19.02	0.088
	Ksh 5,000				
	Ksh 5,000–20,000	101	36.5%		
	Ksh 20,000–50,000	51	18.4%		
	Above Ksh 50,000	18	6.5%		
**Marital Status**	Married	155	55.96%	3.79	0.875
	Single	75	27.08%		
	Widowed	47	16.97%		
**Social Support**	No	245	88.45%	2.08	0.721
	Yes	32	11.55%		
**Hospital**	Hospital 1	92	33.21%	4.08	0.942
	Hospital 2	92	33.21%		
	Hospital 3	93	33.57%		

### Individual Factors Associated with Patients' Satisfaction with Healthcare Services

The survey showed that 60.7% of respondents had confidence in healthcare providers, 60.3% sought care promptly, and 61.4% reported effective communication (Table 3). However, 48.7% struggled with affordability and 44.8% cited unreasonable waiting times. Logistic regression confirmed communication as the strongest predictor of satisfaction (B = 0.857, p = 0.0008, OR = 2.357), while trust (B = 0.236, p = 0.14) and waiting time (B = 0.191, p = 0.079) were non-significant. Understanding (B = −0.163, p = 0.196) and affordability (B = −0.121, p = 0.419) had weak negative effects (Table 4). Communication remains pivotal in shaping patient satisfaction.

**Table 3:** Association Between Individual Factors and Patient Satisfaction

Question	SA (5)	A (4)	NS (3)	D (2)	SD (1)	Chi-square (X^2^)	p-value
How well do you understand your medical condition and treatment plan?	76 (27.4%)	84 (30.3%)	51 (18.4%)	39 (14.1%)	27 (9.7%)	**8.63**	**0.079**
Have your past experiences with healthcare services been positive?	69 (24.9%)	94 (33.9%)	65 (23.5%)	37 (13.4%)	12 (4.3%)	**5.12**	**0.276**
How much trust do you have in healthcare providers to deliver quality care?	77 (27.8%)	91 (32.9%)	50 (18.1%)	32 (11.6%)	27 (9.7%)	**9.84**	**0.041**
How would you rate your overall health status?	45 (16.2%)	76 (27.4%)	79 (28.5%)	48 (17.3%)	29 (10.5%)	**6.45**	**0.198**
Did the hospital services meet your expectations?	58 (20.9%)	103 (37.2%)	63 (22.7%)	32 (11.6%)	21 (7.6%)	**7.33**	**0.092**
Are you able to afford the cost of healthcare services?	35 (12.6%)	52 (18.8%)	55 (19.9%)	67 (24.2%)	68 (24.5%)	**4.89**	**0.303**
Do you seek medical care as soon as you notice symptoms?	83 (30.0%)	84 (30.3%)	42 (15.2%)	50 (18.1%)	18 (6.5%)	**10.27**	**0.037**
Do you feel comfortable discussing your health concerns with doctors?	73 (26.4%)	96 (34.7%)	65 (23.5%)	27 (9.7%)	16 (5.8%)	**11.68**	**0.022**
Was the waiting time at the hospital reasonable?	54 (19.5%)	41 (14.8%)	58 (20.9%)	82 (29.6%)	42 (15.2%)	**13.44**	**0.009**
How satisfied are you with communication between you and your healthcare provider?	87 (31.4%)	83 (30.0%)	59 (21.3%)	27 (9.7%)	21 (7.6%)	**15.89**	**0.004**

**Table 4:** Regression Analysis for Individual Factors Associated with Patient Satisfaction

Variable	Estimate (B)	Std. Error	Wald	Sig. (p-value)	Odds Ratio (Exp(B))
How well do you understand your medical condition and treatment plan?	-0.163	0.126	1.67	0.196	0.849
Have your past experiences with healthcare services been positive?	-0.036	0.238	0.02	0.878	0.964
How much trust do you have in healthcare providers to deliver quality care?	0.236	0.16	2.18	0.14	1.266
How would you rate your overall health status?	-0.071	0.123	0.33	0.565	0.932
Did the hospital services meet your expectations?	0.117	0.151	0.6	0.438	1.124
Are you able to afford the cost of healthcare services?	-0.121	0.149	0.65	0.419	0.886
Do you seek medical care as soon as you notice symptoms?	0.164	0.118	1.93	0.165	1.178
Do you feel comfortable discussing your health concerns with doctors?	-0.068	0.154	0.19	0.661	0.935
Was the waiting time at the hospital reasonable?	0.191	0.109	3.08	0.079	1.211
How satisfied are you with communication between you and your healthcare provider?	0.857	0.255	11.32	0.0008	2.357

### Individual Factors Associated with Patients' Satisfaction with Healthcare Services

The survey showed that 60.7% of respondents had confidence in healthcare providers, 60.3% sought care promptly, and 61.4% reported effective communication (Table 3). However, 48.7% struggled with affordability and 44.8% cited unreasonable waiting times. Logistic regression confirmed communication as the strongest predictor of satisfaction (B = 0.857, p = 0.0008, OR = 2.357), while trust (B = 0.236, p = 0.14) and waiting time (B = 0.191, p = 0.079) were non-significant. Understanding (B = −0.163, p = 0.196) and affordability (B = −0.121, p = 0.419) had weak negative effects (Table 4). Communication remains pivotal in shaping patient satisfaction. Regression analysis revealed that communication with healthcare providers was the strongest predictor of satisfaction (B = 0.857, p = 0.0008, OR = 2.357), with patients who reported better communication being more than twice as likely to be satisfied (Table 5). Waiting time (B = 0.191, p = 0.079, OR = 1.211) and trust (B = 0.236, p = 0.14, OR = 1.266) showed positive but non-significant associations. Understanding of conditions (B = −0.163, p = 0.196), affordability (B = −0.121, p = 0.419), and prior experiences (B = −0.036, p = 0.878) did not predict satisfaction. These findings underscore communication as a critical driver of patient experience (Table 4).

## Discussion

The study aimed to examine the extent and determinants of patient satisfaction within Level 5 public hospitals in Kiambu County. Findings indicated a moderate level of satisfaction, with considerable variation in individual experiences, suggesting that the health system was perceived as functional yet constrained by service delivery inconsistencies. While many patients expressed confidence in the care they received, dissatisfaction persisted in areas such as interpersonal communication, drug availability, and institutional responsiveness. These patterns reflected challenges commonly observed in public health facilities across low- and middle-income countries, where growing expectations often collided with systemic limitations^21^–^23^. Within this context, patient satisfaction emerged as a complex construct shaped by relational, institutional, and structural elements of care^2^,^23^,^24^. These findings underscored the importance of examining how contextual realities shaped patient experiences within constrained service environments^25^. Socio-demographic variables such as age, gender, education, and income did not significantly influence satisfaction, reaffirming prior findings that personal attributes were often secondary to health system factors in shaping perceptions of care^22^,^26^. Although some studies suggested demographic effects, particularly in settings where privatized options widened disparities^27^,^28^, the uniformity in public sector service provision likely diluted such distinctions. Employment status, however, showed a borderline association with satisfaction, a finding consistent with evidence linking economic security to healthcare access, decision-making confidence, and reduced stress^21^,^29^. These results highlighted that while demographic profiles had limited predictive value, broader social determinants—particularly employment—played an important role in shaping healthcare experiences, especially in systems where user costs and indirect expenses affected accessibility. Among individual-level factors, communication with healthcare providers emerged as the most powerful predictor of patient satisfaction. Patients who reported respectful, clear, and responsive communication were significantly more likely to express satisfaction, reaffirming prior studies that had positioned communication at the core of therapeutic relationships^23^,^24^,^30^. In resource-constrained health systems, where gaps in infrastructure and supplies persisted, provider communication often served as a proxy for perceived quality and institutional commitment^23^,^31^. This study reinforced that communication was not simply a conduit for information but a relational determinant that shaped trust, agency, and emotional safety within clinical encounters. These findings supported the integration of communication skills into clinical training and underscored the need for institutional cultures that valued relational competence alongside clinical efficiency, especially in high-volume public facilities.

Other individual factors, including understanding of treatment, affordability of care, trust in providers, and previous health experiences, did not demonstrate significant associations with satisfaction. While trust and perceived waiting times showed positive trends, they did not reach statistical significance in this study. These findings diverged from literature that had previously linked affordability and provider trust to satisfaction^21^,^22^,^27^, and suggested that the influence of these variables might be context-dependent. The predominance of communication in shaping satisfaction may have overshadowed the effects of other personal factors. This interpretation aligned with previous research suggesting that communication often mediated or amplified the perceived effects of structural or clinical deficits^24^,^31^. The results emphasized that while structural improvements remained necessary, relational engagement between patients and providers had the potential to meaningfully elevate patient experiences.

The study's findings carried significant implications for health policy and service delivery in Kenya. As the country advanced toward Universal Health Coverage, patient satisfaction needed to be repositioned from a supplementary metric to a core indicator of quality and accountability^21^,^32^. The observed patterns underscored the importance of integrating patient-centered care into public health reform efforts. Routine collection and use of patient feedback, continuous professional development in communication, and structural reforms aimed at reducing waiting times and improving medication access were all necessary to align service delivery with patient expectations. Furthermore, this study demonstrated that improving satisfaction required both systemic and relational reforms. A shift was required from service models that emphasized throughput and compliance to those that valued human connection, dignity, and mutual respect—cornerstones of ethical, high-quality healthcare in any context^23^,^24^,^30^.

## Limitations

The study was limited to three hospitals in Kiambu county, This may not represent the experiences of patients in other counties. Future studies could include a broader sample of hospitals.

## Conclusion

This study concluded that patient satisfaction with outpatient services in Level 5 public hospitals in Kiambu County was moderate, with communication between healthcare providers and patients emerging as the most significant determinant. Socio-demographic variables, aside from employment status, were not strongly associated with satisfaction, underscoring the centrality of interpersonal care and system responsiveness in shaping patient perceptions. Based on these findings, it is recommended that healthcare providers undergo continuous professional development in communication and patient-centered care. Health facilities should institutionalize routine patient feedback mechanisms to enhance service responsiveness and accountability. Policy-makers must also prioritize reducing systemic inefficiencies such as drug stockouts and prolonged wait times. Integrating satisfaction metrics into routine performance monitoring within the Universal Health Coverage framework could promote a culture of quality improvement. Collectively, these efforts can enhance patient experience, rebuild trust in public healthcare, and advance the broader goal of delivering equitable and person-centered care in Kenya's devolved health system.
